# Uncovering the clinical relevance of unclassified variants in DNA repair genes: a focus on *BRCA* negative Tunisian cancer families

**DOI:** 10.3389/fgene.2024.1327894

**Published:** 2024-01-19

**Authors:** Maroua Boujemaa, Fatma Nouira, Nouha Jandoubi, Nesrine Mejri, Hanen Bouaziz, Cherine Charfeddine, Sonia Ben Nasr, Soumaya Labidi, Houda El Benna, Yosra Berrazega, Haifa Rachdi, Nouha Daoud, Farouk Benna, Abderrazek Haddaoui, Sonia Abdelhak, Mohamed Samir Boubaker, Hamouda Boussen, Yosr Hamdi

**Affiliations:** ^1^ Laboratory of Biomedical Genomics and Oncogenetics, LR20IPT05, Institut Pasteur de Tunis, University of Tunis El Manar, Tunis, Tunisia; ^2^ Laboratory of Bioactive Substances, Center of Biotechnology of Borj Cedria, University of Tunis El Manar, Hamam, Tunisia; ^3^ Medical Oncology Department, Abderrahman Mami Hospital, Faculty of Medicine Tunis, University Tunis El Manar, Tunis, Tunisia; ^4^ Surgical Oncology Department, Salah Azaiez Institute of Cancer, Tunis, Tunisia; ^5^ High Institute of Biotechnology of Sidi Thabet, Biotechpole of Sidi Thabet, University of Manouba, Ariana, Tunisia; ^6^ Department of Medical Oncology, Military Hospital of Tunis, Tunis, Tunisia; ^7^ Radiation Oncology Department, Salah Azaiez Institute, Tunis, Tunisia; ^8^ Laboratory of Human and Experimental Pathology, Institut Pasteur de Tunis, Tunis, Tunisia

**Keywords:** variants of uncertain significance, clinical relevance, DNA repair genes, breast cancer, pathogenicity predictions, segregation analysis

## Abstract

**Introduction:** Recent advances in sequencing technologies have significantly increased our capability to acquire large amounts of genetic data. However, the clinical relevance of the generated data continues to be challenging particularly with the identification of Variants of Uncertain Significance (VUSs) whose pathogenicity remains unclear. In the current report, we aim to evaluate the clinical relevance and the pathogenicity of VUSs in DNA repair genes among Tunisian breast cancer families.

**Methods:** A total of 67 unsolved breast cancer cases have been investigated. The pathogenicity of VUSs identified within 26 DNA repair genes was assessed using different *in silico* prediction tools including SIFT, PolyPhen2, Align-GVGD and VarSEAK. Effects on the 3D structure were evaluated using the stability predictor DynaMut and molecular dynamics simulation with NAMD. Family segregation analysis was also performed.

**Results:** Among a total of 37 VUSs identified, 11 variants are likely deleterious affecting *ATM, BLM, CHEK2, ERCC3, FANCC, FANCG, MSH2, PMS2 and RAD50* genes. The *BLM* variant, c.3254dupT, is novel and seems to be associated with increased risk of breast, endometrial and colon cancer. Moreover, c.6115G>A in *ATM* and c.592+3A>T in *CHEK2* were of keen interest identified in families with multiple breast cancer cases and their familial cosegregation with disease has been also confirmed. In addition, functional *in silico* analyses revealed that the *ATM* variant may lead to protein immobilization and rigidification thus decreasing its activity. We have also shown that *FANCC* and *FANCG* variants may lead to protein destabilization and alteration of the structure compactness which may affect FANCC and FANCG protein activity.

**Conclusion:** Our findings revealed that VUSs in DNA repair genes might be associated with increased cancer risk and highlight the need for variant reclassification for better disease management. This will help to improve the genetic diagnosis and therapeutic strategies of cancer patients not only in Tunisia but also in neighboring countries.

## 1 Introduction

Genetic testing of hereditary breast cancer has become broadly available especially with the emergence of Next Generation Sequencing (NGS) technologies that have revolutionized the field of genomics (Yadav and Couch, 2019). To identify women at increased risk of developing breast cancer, the National Comprehensive Cancer Network and the American Society of Clinical Oncology both recommend complete mutation screening of *ATM, CDH1, CHEK2, PALB2, PTEN, STK11* and *TP53* in addition to *BRCA1/2* genes*.* There is also accumulative evidence that mutations in *BARD1, BRIP1, MSH2, MLH1, MSH6, NBN, NF1, PMS2, RAD51C,* and *RAD51D* predispose to hereditary breast cancer (Urbina-Jara et al., 2019; Yadav and Couch, 2019). Carriers of risk variants may benefit from enhanced screening, chemoprevention and/or preventive surgery. Genetic testing is also becoming increasingly relevant in cancer therapy since patients with defects in DNA repair genes particularly those with *BRCA* mutations and/or with Homologous Recombination Deficiency (HRD) are more sensitive to platinum-based therapies and may also benefit from PARP inhibitors (Monteiro et al., 2020; Gonzalez and Stenzinger, 2021). Certainly, that progress made in NGS technologies has increased our capability to understand the genetic basis of cancer (Gerasimavicius et al., 2020). Nevertheless, the main challenge in clinical genetic testing remains the interpretation of the identified variants particularly those with uncertain significance (Ernst et al., 2018; Gerasimavicius et al., 2020; Monteiro et al., 2020). Disease causing variants are usually nonsense mutations that result in a premature stop codon, small indels causing a frameshift and a premature termination of translation, splicing site mutations that occur inside of the canonical splice sites, large deletions or known deleterious missense variants. Whereas variants with insufficient or conflicting evidence of pathogenicity that support their association with disease, are classified as Variants of Uncertain Significance (VUSs). These variants are usually missense substitutions and small in-frame indels (Girolimetti et al., 2014; Joynt et al., 2022). In public databases more than 90% of missense variants identified by clinical genetic testing are described as variants of uncertain significance (Girolimetti et al., 2014; Landrum et al., 2020), of these a significant fraction is identified within *BRCA* genes and other DNA repair genes involved in cancer predisposition (Hart et al., 2019; Monteiro et al., 2020). The lack of evidence on the pathogenicity of VUSs in these cancer associated genes represent a critical clinical challenge and efforts to review the classification of these variants are urgently needed. With this in mind, several initiatives have been established such as “ENIGMA consortium” aiming to determine the clinical significance of sequence variants in *BRCA1*, *BRCA2* and other known or candidate breast cancer susceptibility genes (Spurdle et al., 2012; Richards et al., 2015; Hart et al., 2019; Heczkova et al., 2019). Moreover, the genetic and cancer group (GGC) – Unicancer have launched the COVAR project in order to classify the maximum of VUSs, detected in *BRCA1/2* and in genes included in hereditary cancer panels*,* in terms of their probability to be pathogenic based on co-segregation analysis (Cohen-Haguenauer, 2019).

In the current report, we aim to assess the clinical relevance and the pathogenicity of VUSs in DNA repair genes in Tunisian cancer families.

## 2 Materials and methods

### 2.1 Patients

A total of 67 unsolved hereditary breast cancer cases were included in this study. Written informed consents were obtained from all participants. The study was conducted according to the Declaration of Helsinki Principles and ethical approval was obtained from the biomedical ethics committee of Institut Pasteur de Tunis (2017/16/E/Hopital A-M & 2019/1/I/LR16IPT05).

### 2.2 DNA isolation

Total genomic DNA was isolated from peripheral blood using QIAamp DNA Mini Kit (Qiagen) according to the manufacturer’s instructions. DNA purity and concentration were measured using a NanoDrop™ spectrophotometer.

### 2.3 Next Generation Sequencing and data analysis

Targeted sequencing using a panel of 24 genes was performed on 53 patients. This panel included the following genes: *BRCA1, BRCA2, PALB2, CDH1, PTEN, TP53, RAD51C, RAD51D, MLH1, MSH2, MSH6, PMS2, EPCAM, ATM, BRIP1, CHEK2, STK11, MRE11A, NBN, RAD50, BARD1, BLM, XRCC2* and *MUTYH.* Target enrichment of coding regions and intron/exon junctions (±50bp) was performed by Twist technology followed by a pair-end sequencing reaction (2 × 150bp) on a Nextseq2000 platform (Illumina).

Whole Exome Sequencing was carried out on 14 patients. Samples were prepared according to Agilent SureSelectXT Human All Exon V6 Protocol and enrichment was done according to Agilent SureSelect protocols. Paired-end (2 × 150) sequencing was performed on enriched samples on the Illumina NovaSeq6000 system.

Bioinformatic analysis was performed using an In-House pipeline. Data quality control and preprocessing (including adapter trimming, quality trimming and removal of very short reads) were performed using FastQC (https://www.bioinformatics.babraham.ac.uk/projects/fastqc/) and BBDuk (https://jgi.doe.gov/data-and-tools/software-tools/bbtools/) tools respectively. DNA sequences were mapped to their location in the build of the human genome (hg19/b37) using the Burrows–Wheeler Aligner (BWA) package (Li and Durbin, 2009). The subsequent SAM files were converted to BAM files using Samtools (Li et al., 2009). Duplicate reads were removed using Picard version 2.6 (http://broadinstitute.github.io/picard/). Post-Alignment quality control and variant calling were performed using GATK version 4.1.2 (McKenna et al., 2010). VarAFT version 2.16 was subsequently used for variant annotation (Desvignes et al., 2018). First, we have looked for known pathogenic variants in 37 genes frequently analyzed in high-risk breast and ovarian cancer families (Suszynska et al., 2019). Then, we have focused our analysis on a set of DNA repair genes to unravel the genetic etiology of the investigated cases.

### 2.4 DNA repair genes investigation

In order to evaluate whether variants in DNA repair genes might be associated with hereditary predisposition in the Tunisian population, a set of 169 genes (Supplementary Table S1) have been investigated. This list included known genes associated with hereditary predisposition to cancer in addition to other genes belonging to 7 major DNA repair pathways retrieved from KEGG GENES Database (https://www.genome.jp/kegg/genes.html) and mdanderson data (https://www.mdanderson.org/documents/Labs/Wood-Laboratory/human-dna-repair-genes.html) and it was limited to 24 genes for patients investigated by gene panel. The 7 major DNA repair pathways are as follows: 1) Base-excision repair (BER) 2) Mismatch repair (MMR) 3) Nucleotide-excision repair (NER) 4) Homologous recombination (HR) 5) Nonhomologous end joining (NHEJ) 6) Translesional synthesis (TLS) and 7) Fanconi Anemia pathways. Among these genes, unclassified genetic variants described as variants of uncertain significance, having conflicting interpretations of pathogenicity in the ClinVar database or not described in public databases were selected for further analyses.

Protein-protein interaction network and functional enrichment analyses were performed using String (Jensen et al., 2009) and EnrichR databases (Kuleshov et al., 2016).

### 2.5 Assessment of the functional impact of variants

#### 2.5.1 Pathogenicity predictions (sequence based prediction)

The pathogenicity of variants was assessed using several *in silico* predictions tools including: SIFT (Vaser et al., 2016), PolyPhen2 (Adzhubei et al., 2013), LRT (Chun and Fay, 2009), MutationAssessor (Reva et al., 2011) PROVEAN (Choi et al., 2012), MutationTaster (Schwarz et al., 2010), FATHMM (Rogers et al., 2018), CADD (Kircher et al., 2014), MetaLR/MetaSVM (Dong et al., 2015), UMD-predictor (Salgado et al., 2016) and Align-GVGD (Tavtigian et al., 2006).

In addition, we have used VarSEAK (https://varseak.bio/), SpliceAI Lookup (https://spliceailookup.broadinstitute.org/) and Human Splicing Finder (HSF) (Desmet et al., 2009) to predict the effect of variants on splicing events. TraP v3 (Gelfman et al., 2017) and FATHMM-XF (Rogers et al., 2018) were used to predict the pathogenicity of synonymous as well as non-coding variants.

#### 2.5.2 *In-silico* functional analyses (structure based prediction)

*In silico* functional analyses were performed to evaluate the impact of the identified variants on the protein structure. Experimentally derived three-dimensional protein structures were extracted from the Protein Data Bank (PDB) archive which represents the single repository of information about the 3D structures of proteins, nucleic acids, and complex assemblies (Berman et al., 2000). HHpred server (Soding et al., 2005) in combination with MODELLER software (Webb and Sali, 2016) were employed for structure prediction and homology modeling by using the MPI Bioinformatics Toolkit (Gabler et al., 2020) (https://toolkit.tuebingen.mpg.de). The impact of mutations on protein stability and flexibility was assessed using the DynaMut web server which also provides inter-residue interactions for both wild-type and mutant structures (Rodrigues et al., 2018). Stability and flexibility are evaluated respectively through the estimation of the difference in ΔΔG and ΔΔSVib between the normal and the mutant protein. The ΔΔG represents the folding free energy of the protein and is expressed in kcal/mol. DynaMut defines mutations with ΔΔG ≥0 as Stabilizing and those with ΔΔG <0 as deStabilizing. The effect on protein stability is considered as significant if ΔΔG ≥0.5 (Stabilisation) or ΔΔG < −0.5 (destabilization). The ΔΔSVib or vibrational entropy energy contributes significantly to protein binding free energies. DynaMut implements ENCoM, to calculate ΔΔSVib as the difference between the vibrational energy of the wild-type and mutant structures. A ΔΔSVib <0 represents a rigidification of the protein structure while a ΔΔSVib ≥0 represents a gain in flexibility. To enhance the reliability of our results, we have also used the FoldX plugin to predict the effects of mutations on protein stability (Van Durme et al., 2011). Furthermore, a molecular dynamics simulation was performed using the two software packages VMD (Humphrey et al., 1996) and NAMD (Phillips et al., 2020) to better understand the effects of the identified variants on the 3D structure of the protein. Different parameters have been studied all over the simulation trajectory including root mean square deviation (RMSD) and the radius of gyration (Rg).

## 3 Results

**FIGURE 1:**
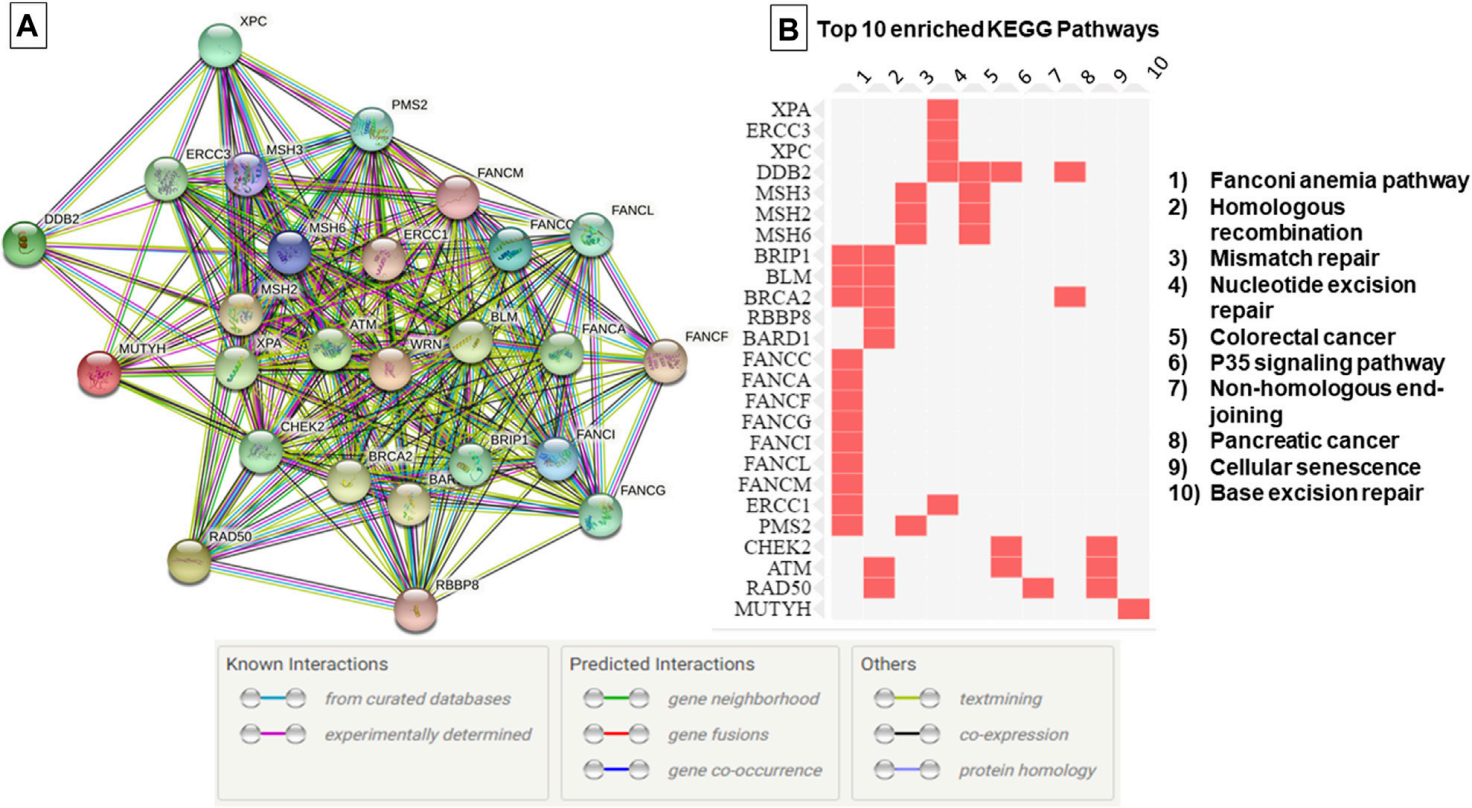
Protein-Protein interaction network and top 10 enriched pathways according to the KEGG database. **(A)** Protein-Protein interaction network generated using String database, **(B)** Enriched pathways as generated by EnrichR database based on data from KEGG PATHWAY. The edges indicate both functional and physical protein associations, line color indicates the type of interaction evidence.

The genetic investigation in 67 breast cancer cases using TGS and WES showed the absence of know pathogenic variants in genes associated with hereditary predisposition to cancer. To assess whether novel mutations or misclassified variants are responsible for disease susceptibility a set of 169 DNA repair genes have been investigated. This revealed a total of 37 unclassified variants localized within 26 DNA repair genes (Supplementary Table S2, Figure 1). It is noteworthy that the number of unclassified variants detected appears to be relatively few when compared to the total number of genes investigated. This observation is expected given the fact that most patients were subjected to TGS. In the current study, most of the identified variants were exonic (62%), 11% were splicing [±10bp from the exon-intron junction) and the remaining variations intronic and/or located within 5′ and 3′ untranslated regions (Supplementary Figure S1)]. According to the KEGG database, most of the identified variants are localized within genes involved in Fanconi anemia pathway followed by Homologous recombination and Mismatch repair pathway (Figure 1). Identified variants were prioritized based on frequency in public databases and predicted pathogenicity. Only those with a MAF <0.01 in gnomAD database and predicted as deleterious by at least 6 *in silico* tools and/or predicted to alter the protein structure and function were kept for further analyses.

### 3.1 Variant distribution and prioritization

#### 3.1.1 Exonic variants

We have identified 23 exonic variants among which 9 are of particular interest, predicted as deleterious by at least 6 *in silico* prediction tools and/or predicted to affect the protein function (Table 1). They include 6 missense variants localized within *ATM, ERCC3, FANCC, FANCG, MSH2* and *PMS2* genes, 1 novel frameshift deletion in the *BLM* gene, 1 novel in-frame deletion in *CHEK2* gene and 1 frameshift deletion in *PMS2*.

**TABLE 1:** Relevant variants likely associated with cancer predisposition.

Gene	cDNA change	Proteine change	ClinVar	Frequency (GnomAD Exome)	Align GVGD^a^	CADD^b^	Mutaton taster	Mutation assessor	Polyphen2	PROVEAN	SIFT
*ATM*	c.6115G>A	Glu2039Lys	Conflicting interpretations of pathogenicity	0.000004	C55	26.2^b^	D^c^	M^d^	D^e^	D^f^	D^g^
*BLM*	c.3254dupT	Arg1086Lysfs^a^7	Not reported	unknown	—	—	D	—	—	—	D
*CHEK2*	c.493_498delGAATAT	Glu165_Tyr166del	Not reported	unknown	—	—	—	—	—	—	—
*ERCC3*	c.2111C>T	Ser704Leu	Conflicting interpretations of pathogenicity	0.002196	C65	24	D	M	P^h^	D	D
*FANCC*	c.1156T>C	Ser386Pro	Conflicting interpretations of pathogenicity	0.000553	C65	25.4	D	M	D	D	D
*FANCG*	c.366G>C	Trp122Cys	Conflicting interpretations of pathogenicity	0.00080	C65	31	D	M	D	D	D
*MSH2*	c.728G>A	Arg243Gln	Uncertain significance	0.00002	C35	34	D	M	D	D	D
*PMS2*	c.1004A>T	Asn335Ile	Uncertain significance	0.00025	C65	33	D	H^i^	D	D	D
*PMS2*	c.2186_2187del	Leu729Glnfs^a^6	Uncertain significance	0.0018	—	—	D	—	—	—	D

^a^ C55-C65 Most likely affecting function.

^b^ CADD cutoff on deleteriousness 15.

^c^ Disease_causing.

^d^ Medium.

^e^ Probably damaging.

^h^ Possibly damaging.

^f^ Deleterious.

^g^ Deleterious.

^i^ High.

BC, Breast Cancer; CC, Colorectal Cancer; ED, Endometrial Cancer; PC, Prostate Cancer.

#### 3.1.2 Splicing variants

In the current report 4 splicing variants, located ±10bp from the exon-intron junction, have been identified (Supplementary Table S2) among which 2 variants, c.592+3A>T in *CHEK2* and c.3036+5G>A in *RAD50* gene, were predicted to alter the wild-type donor site and to probably affect splicing.

#### 3.1.3 Non-coding variants

Ten non-coding variants have been identified. Among these variants, 4 were rare (MAF <0.01) and *in silico* predictions have not revealed any significant pathogenic effect (Supplementary Table S2).

In summary, among all the unclassified variants identified in this study, 11 are likely the most relevant (9 coding and 2 splicing).

### 3.2 Genotype phenotype correlation and segregation analysis

A total of 11 variants likely associated with hereditary predisposition have been identified and are localized within *ATM, BLM, CHEK2, ERCC3, FANCC, FANCG, MSH2, PMS2* and *RAD50* genes.

**FIGURE 2:**
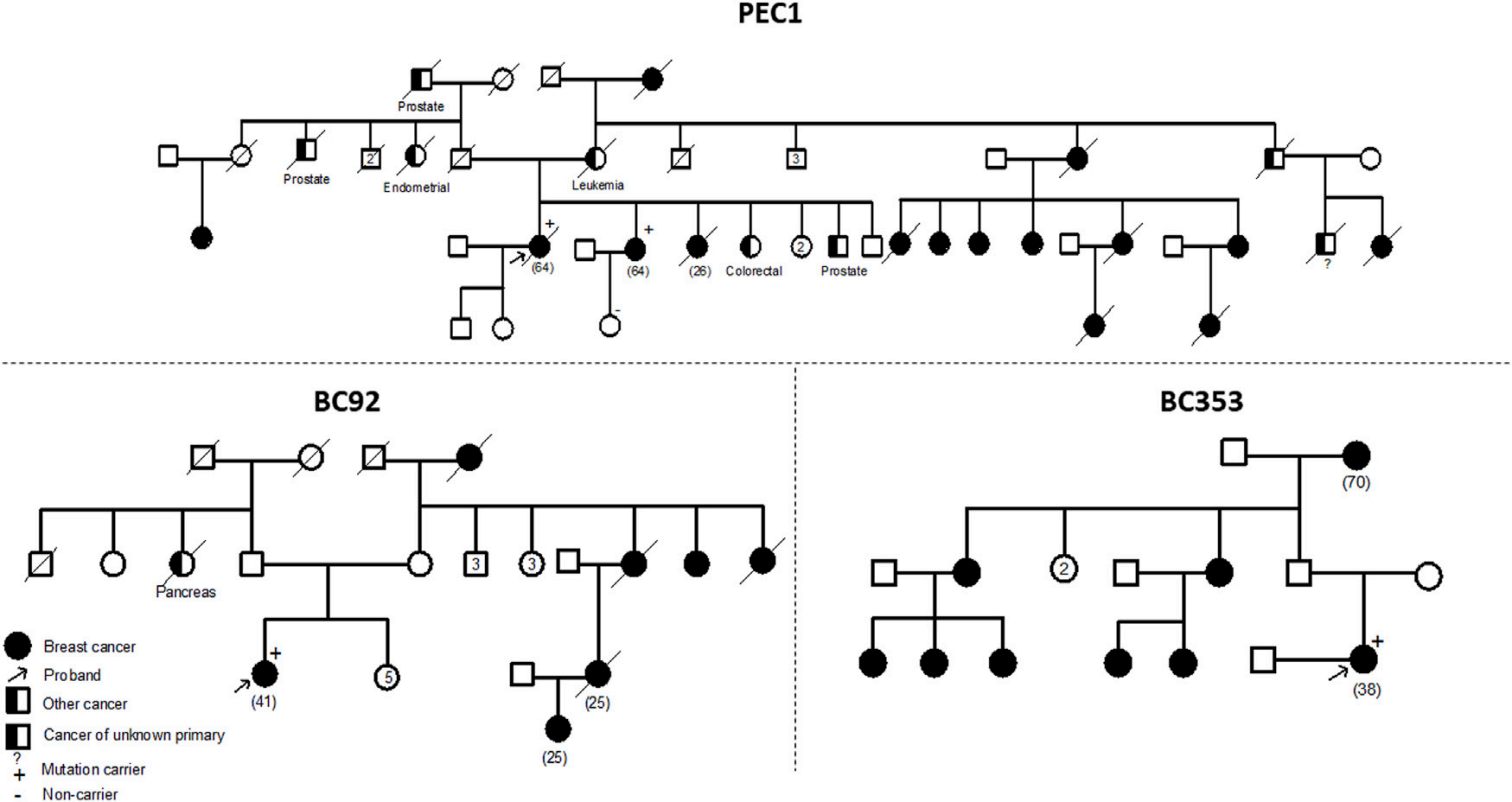
Pedigrees of families carriers of the ATM c.6115G>A variant. Confirmation of the segregation of c.6115G>A, in *ATM* gene, with disease. This variant was identified in 4 affected cases belonging to 3 families and was absent in one healthy tested relative. A strong family history of breast cancer was noteworthy observed in the 3 families. Age at diagnosis (if available) is stated between brackets.

The *ATM* variant, c.6115G>A, results in the change of a Glutamic Acid to a Lysine. It was identified in 2 unrelated patients belonging to the same geographical region. A strong family history of breast cancer was observed among these two cases. The first patient is 41 years old with 6 cases of breast cancer in the family and the second is 38 years old having 8 cases of breast cancer in the family. A targeted mutation screening was conducted in this geographical region and allowed the identification of a third carrier who had developed breast cancer at the age of 64. This variation was subsequently confirmed in her sister diagnosed also with breast cancer and was absent in the healthy niece. In this family, a strong family history of breast cancer was also observed where 14 breast cancer cases were detected. These findings support the pathogenicity of this variation and suggest a possible founder effect as well as a plausible association with a breast cancer only phenotype (Figure 2).

**FIGURE 3:**
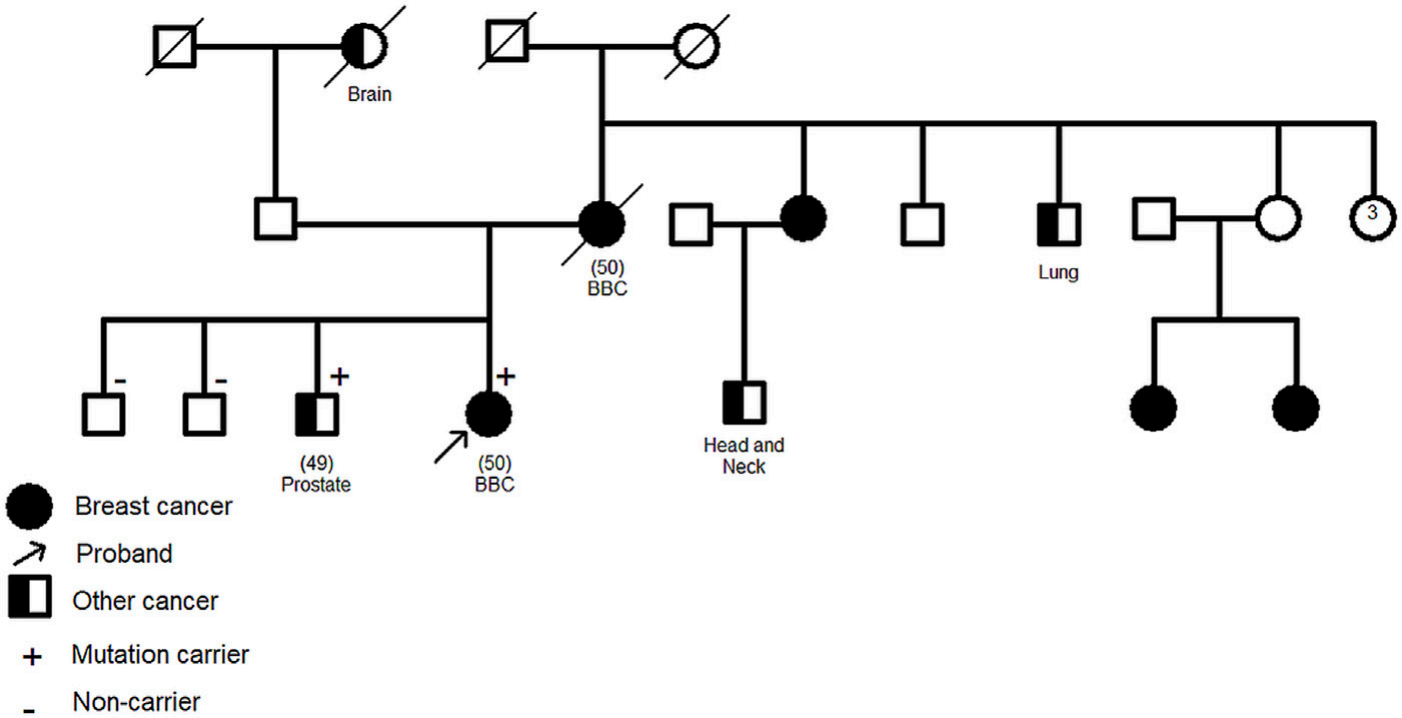
Family pedigree of c.592+3A>T CHEK2 carriers. Confirmation of the segregation of c.592+3A>T, in *CHEK2* gene, with disease. It was identified in two affected cases (1 breast cancer, 1 prostate cancer) and was absent in healthy siblings. Age at diagnosis (if available) is stated between brackets. BBC: Bilateral Breast Cancer.

The *CHEK2* variant, c.592+3A>T, consists of an A>T nucleotide substitution at the +3 position of intron 4 of the *CHEK2* gene. *In silico* predictions revealed that this variant may alter the wild type of the donor site leading to exon skipping (Table 2). It was identified in a 50 year old woman diagnosed with bilateral breast cancer. This same mutation was present in her brother diagnosed with prostate cancer at 49 years old and was absent in healthy siblings (Figure 3) which support the pathogenicity of this variant.

**TABLE 2:** Splice sites variations likely associated with disease predisposition.

Variation	Carriers	Disease	VarSEAK	Human splicing finder	SpliceAI lookup
NM_007194.4(*CHEK2*): c.592+3A>T	BC419-1	BC	Class 5: Splicing effect	Broken WT Donor Site: Alteration of the WT Donor site, most probably affecting splicing	High probability of Donor site loss
	BC419-2	PC	Loss of function for authentic Splice Site: Exon Skipping		
			VarSEAK Classification: Likely Pathogenic		
NM_005732.4(*RAD50*): c.3036+5G>A	BC40	BC	Class 4: Likely splicing effect	Broken WT Donor Site: Alteration of the WT Donor site, most probably affecting splicing	No Splicing Effect
			Likely loss of function for authentic Splice Site. Exon Skipping		

BC, Breast Cancer; PC, Prostate Cancer.

For the other identified variants, segregation analysis was not accessible due to the fact that most of the affected members of the investigated families were deceased at the time of the analysis. Among these high-risk variants, one novel homozygous frameshift deletion was identified in the *BLM* gene, c.3254dupT, that leads to premature termination of translation at codon position 1092. Interestingly, this variant was detected in one breast cancer patient with a personal history of endometrial and colorectal cancer. No other affected cases were identified within the family. *BLM* gene mutations are known to be associated with increased risk of developing multiple cancers including breast (Kluzniak et al., 2019), colorectal cancers (de Voer et al., 2015) and endometrial cancer (Long et al., 2019) which is consistent with our findings and support the pathogenicity of the identified variant.

Another novel deletion c.493_498delGAATAT was detected in the *CHEK2* gene. This in-frame deletion is located in the exon 4 of *CHEK2* gene and predicted to lead to the loss of two amino acids, Glu and Tyr in positions 165 and 166 of the protein. This deletion lies within the forkhead-associated (FHA) domain which is critically involved in dimerization of CHK2 molecules in phosphorylation-dependent manner. The patient harboring this variant was diagnosed with RH-/HER2+ breast cancer at 27 years old. Family history of early onset breast cancer was also observed among relatives in addition to other types of cancer.

Among the selected variants, c.728G>A in *MSH2* gene was identified in one breast cancer case with a family history of ovarian, gastric and lung cancers. Interestingly, this same variant was recently described in a Tunisian patient with gastric cancer that has a family history of breast, ovarian, and colon cancers (Kabbage et al., 2022). In this same study, structural bioinformatics analyses revealed that this variant is involved in the MSH2-MLH1 complex stability and may impact on the binding of MSH2 protein with MLH1 by disrupting the electrostatic potential which is suggestive of a pathogenic effect.

Moreover, one breast cancer patient (BC310) with family history of stomach, colon and breast cancer, harbored a frameshift deletion in the *PMS2* gene (c.2186_2187del). This latter is classified as a variant of uncertain significance in the ClinVar database although it is predicted to result in the premature termination of the protein at amino acid position 734 leading to protein function alteration. Genetic data and family history of BC310 are consistent with Lynch syndrome which is known to be associated with an increased risk of developing colorectal cancer as well as other tumor types including breast and gastric cancers (Fornasarig et al., 2018; Sheehan et al., 2020). In the same gene, a missense variation, Asn335Ile, was identified in a patient diagnosed with early onset breast cancer and with family history of breast, skin and pancreatic cancers. Considering *ERCC3* gene, a missense variation Ser704Leu was identified in a breast cancer patient with multiple affected relatives. In the same context, *ERCC3* germline mutations were previously identified in families with multiple breast cancer cases (Vijai et al., 2016). Two missense variations in *FANCC* and *FANCG* genes, predicted to be deleterious, were further identified in breast cancer cases with family history of different tumors including leukemia, breast, prostate, and colon cancers. Finally, a splicing mutation predicted to lead to exon skipping was identified in *RAD50* gene (Tables 2, 3) in a breast cancer patient aged 37 years.

**TABLE 3:** Clinicopathological features of patients carriers of relevant variants.

Variant	Patient ID	Screening method	Pathology	Age at diagnosis	Family history of cancer	Hormone receptor status	HER2 status	Ki67
*ATM* c.6115G>A	BC92	WES	Breast Cancer	41	6 Breast	NA	NA	NA
					1 Pancreas			
	BC353	TGS	Breast Cancer	38	8 Breast	ER+/PR+	HER2-	15%
	PEC1-1	Sanger sequencing	Breast Cancer	64	14 Breast	ER+/PR-	HER2-	NA
	PEC1-2	Sanger sequencing	Breast Cancer	64	2 Prostate	NA	NA	NA
					1 Endometrium			
					1 Colorectal			
					1 Lung			
*BLM* 3254dupT	BC418	TGS	Endometrial Cancer	47	-	NA	NA	NA
			Breast Cancer	48				
			Colorectal Cancer	51				
*CHEK2* c.493_498delGAATAT	BC401	TGS	Breast Cancer	27	1 Breast	ER-/PR-	HER2+	60%
					1 Pancreas			
*CHEK2* c.592+3A>T	BC419-1	TGS	Breast Cancer	50	4 Breast	NA	NA	NA
	BC419-2	Sanger sequencing	Prostate Cancer	49	1 Prostate	-	-	-
					1 Head and neck			
					1 Brain			
					1 Lung			
*ERCC3* c.2111C>T	BC19	WES	Breast Cancer	49	3 Breast	ER-/PR-	NA	NA
*FANCC* c.1156T>C	BC252-1	WES	Breast Cancer	40	2 Prostate	ER+/ER+	HER2-	30%
					3 Breast			
					1 Lung			
					1 Leukemia			
*FANCG* c.366G>C	BC39	WES	Breast Cancer	60	1 Breast	ER+/PR+	HER2-	50%
					1 colon			
					1 Endometrium			
					1 Testes			
*MSH2 c.728G>A*	BC47	WES	Breast Cancer	48	1 Ovarian	ER-/PR-	HER2+	NA
					1 Stomach			
					1 Lung			
*PMS2* c.1004A>T	BC22	WES	Breast Cancer	29	1 Breast	ER+/PR+	NA	NA
					1 Pancreas			
					1 Skin			
*PMS2* c.2186_2187del	BC310	WES	Breast Cancer	55	5 Stomach	NA	NA	NA
					1 Colon			
					1 Breast			
*RAD50 c.3036+5G>A*	BC40	WES	Breast Cancer	37	1 Breast	ER+/PR+	HER2+	22%
					1 Stomach			

### 3.3 *In-silico* functional analyses

**FIGURE 4:**
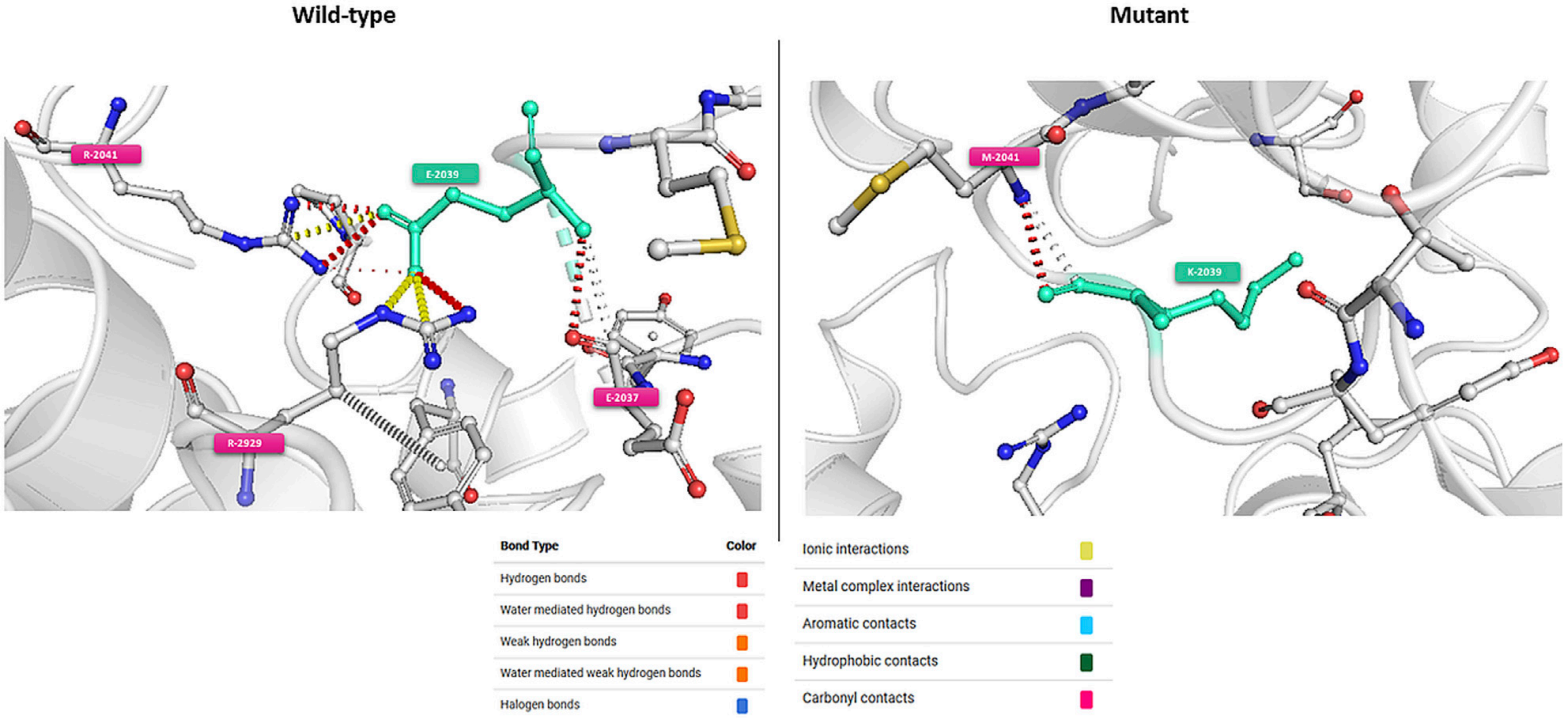
Visual representation of Δ Vibrational Entropy Energy **(A)** and Prediction of Interatomic Interactions **(B)**. Amino acids are colored according to the vibrational entropy change upon mutation. BLUE represents a rigidification of the structure. Wild-type and mutant residues are colored in light-green and are also represented as sticks alongside with the surrounding residues which are involved in any type of interactions.

*In-silico* functional analyses including stability and flexibility analysis as well as molecular dynamics simulations were performed to predict the impact of coding missense variants on the 3D structure of the corresponding proteins mainly ATM, ERCC3, FANCC, FANCG, and PMS2. Among these variants, c.6115G>A (E2039K) in the *ATM* gene is of particular interest since it was identified in families with multiple cases of breast cancer (more than 6 cases). This variant sits within the regulatory FAT domain of the ATM protein. Predictions of protein stability and flexibility changes upon mutation revealed that E2039K may increase protein stability and lead to the rigidification of the protein structure (Table 4; Figure 4). Predicting stability changes using the FoldX plugin was also in line with the DynaMut results revealing an increase in stability upon mutation with a ΔΔG = −29,37 (FoldX provides negative ΔΔG values for stabilizing mutations and positive values for destabilizing mutants). Our findings revealed also that E2039K may result in a change of interatomic molecular interactions with surrounding amino acids consisting of interatomic interactions loss with E2037, R2748 and R2929 and gain of interactions with M2041, as shown in Figure 4. Analysis of molecular dynamics results has also supported the previous findings. Indeed, the RMSD profile showed lower RMSD values of the mutant structure compared to the native protein consistent with a gain in stability. In addition, the radius of gyration (Rg) analysis, which gives information on protein compactness, revealed that the mutant protein is more compact than the native form thus correlating with the stability analysis results (Supplementary Figure S2). Among the other investigated variants, and based on DynaMut predictions, we have found that the amino acid changes S386P in FANCC and W122C in FANCG may lead to protein destabilization associated with a significant increase in flexibility particularly for W122C (FANCG). Furthermore, molecular dynamics simulation results have shown higher RMSD values of the mutant structures due to S386P (FANCC) and W122C (FANCG) compared to native proteins. This decrease in stability correlated with decrease in FANCC compactness for S386P while a high increase in compactness was observed for W122C compared to the native FANCG protein as observed in the Rg plots. The assessment of changes in stability and flexibility upon the amino acid changes S704L in ERCC3 and N335I in PMS2 revealed no significant effects on the protein structure (Table 3). Indeed, Molecular dynamic simulation results showed a slight increase in protein stability and decrease in protein compactness for S704L and N335I (Supplementary Figure S2).

**TABLE 4:** Prediction of the impact of missense variants on protein stability and flexibility.

Variant	Protein	ΔΔG DynaMut (kcal/mol)	ΔΔG ENCoM (kcal/mol)	ΔΔS ENCoM (kcal.mol-1.K-1)
E2039K	ATM	**1.903 (Stabilizing)**	**4.519 (Stabilizing)**	**−4.148 (Decrease of molecule flexibility)**
S704L	ERCC3	0.297 (Stabilizing)	−0.056 (Destabilizing)	0.069 (Increase of molecule flexibility)
S386P	FANCC	**−0.783 (Destabilizing)**	−0.320 (Destabilizing)	0.399 (Increase of molecule flexibility)
W122C	FANCG	−0.284 (Destabilizing)	**−1.032 (Destabilizing)**	**1.290 (Increase of molecule flexibility)**
N335I	PMS2	−0.245 (Destabilizing)	−0.044 (Destabilizing)	0.056 (Increase of molecule flexibility)

Bold values refer to significant effects on protein stability.

For the missense variant identified within the *MSH2* gene, no further investigations were performed, as findings of the study of Kabbage et al. (2022) are suggestive of a likely pathogenic effect.

### 3.4 Variant reclassification

Taking into account *in silico* analysis findings, genotype phenotype correlation and segregation analysis results a novel classification based on the American College of Medical Genetics and Genomics (ACMG) guidelines (Richards et al., 2015) was suggested. This novel classification was submitted in the ClinVar database under the submission ID SUB13085050. It highlights the clinical relevance of *ATM*, *BLM* and *CHEK2* variants for which strong evidence of pathogenicity were obtained. The suggested classification and criteria used to reclassify these variants are detailed in Supplementary Table S3.

## 4 Discussion

DNA damage repair genes play a key role in cancer biology and have critical implications in cancer diagnosis and therapy. Cancer cells are usually deficient in normal DNA repair function which causes their genomic instability. DNA repair deficiency also explains the increased sensitivity of cancer cells to genotoxic agents including ionizing radiation and chemotherapy. It is also well established that cells deficient in homologous recombination DNA repair are hypersensitive to polyADP ribose polymerase (PARP) inhibitors. Therefore, resolving the classification of variants of uncertain significance in DNA repair genes is crucial and will lead to better clinical management of cancer (D’Andrea, 2015). Indeed, in some cases therapeutic management may change dramatically, when a variant is upgraded from VUS to pathogenic or likely pathogenic variant. Variant reclassification might allow clinicians to better counsel patients and make recommendations about appropriate medical care (Chiang et al., 2021). In the current report, 11 variants of uncertain clinical significance in *ATM*, *BLM*, *CHEK2, ERCC3, FANCC, FANCG*, *MSH2*, *PMS2* and *RAD50* genes were the most relevant based on *in silico* functional predictions and segregation analysis. Previous studies have shown that heterozygous carriers of *ATM* gene mutations have a 2-5-fold increased risk of developing breast cancer (Moslemi et al., 2021). In the present study, c.6115G>A *ATM* variant, was identified in 4 breast cancer cases belonging to 3 unrelated families all originating from the South of Tunisia suggesting a possible founder effect. A strong family history of breast cancer was clearly observed among these families and segregation analysis has confirmed the co-segregation of this variant with the disease. The presence of multiple breast cancer cases in proband’s relatives in all the 3 families may also suggest a “breast cancer only phenotype” associated with this variation. This same variation seems to be rare in other populations. Indeed, in a large meta-analysis, this variant was observed in 1/2531 breast cancer cases and was absent in 2245 controls (Tavtigian et al., 2009). *In silico* functional analysis performed in this study revealed substantial structural changes due to this amino acid substitution in the mutant protein which made it more stable, less flexible, and more compact. This may in turn alter the protein function. In fact, proteins are highly dynamic molecules, whose function is basically related to their molecular movements (Rodrigues et al., 2018). Several studies have shown that missense variants can lead to protein dysfunction by affecting their stabilities and interactions with other biomolecules. These variants are thought to be deleterious due to reducing or increasing the stability of the corresponding protein (Chen et al., 2020; Birolo et al., 2021). The negative effect of stabilizing mutations is manifested by the rigidification of cooperative movements of subunits, the deregulation of protein-protein interactions and the activity-stability trade-off which implies that an increase in activity is accompanied by a concomitant decrease in the stability of proteins (Siddiqui, 2017; Gerasimavicius et al., 2020). Accordingly, the over-stability of ATM as well as its rigidification may affect the kinase activity of the protein as well as its interactions with other biological molecules. This may disrupt its key role in repairing DNA double-strand breaks and thus promote the risk of developing cancer. All this evidence, along with familial segregation results and *in silico* functional analyses support the pathogenicity of the *ATM* c.6115G>A variant. Considering *CHEK2* gene, two relevant variants were identified, c.493_498delGAATAT and c.592+3A>T. The c.493_498delGAATAT is a novel variant described for the first time in this study, it was neither reported in public databases nor in published literature. This in-frame deletion leads to the loss of two amino acids. It is localized within the forkhead-associated (FHA) domain that is involved in binding to other downstream phosphorylated proteins. Indeed, it mediates ATM-dependent CHK2 phosphorylation and directing of CHK2 to binding partners such as BRCA1, playing hence a critical role in DNA damage response (Li et al., 2002).

The second variant in *CHEK2*, c.592+3A>T, was identified in one family with both breast and prostate cancer cases and segregation analysis has confirmed the cosegregation of the variant with the disease. This variant was predicted to lead to exon skipping and a recent RNA analysis in the study of Apostolou et al. (2021) has confirmed that it leads to exon 3 skipping. In this same study, it was shown that c.592+3A>T is recurrent and founder mutation in the Greek population rising approximately 35 generations ago (Apostolou et al., 2021). These findings along with our results support the pathogenic effect of this variant and highlight the need for variant reclassification for better risk assessment and patients’ management. Indeed, women with *CHEK2* mutations have a 28%–37% lifetime risk of developing breast cancer. This risk is higher in women with a strong family history of the disease (Moslemi et al., 2021). In male carriers, the risk of prostate cancer is higher given that *CHEK2* upregulation reduces cell growth whereas its downregulation alters androgen receptor activity (Apostolou and Papasotiriou, 2017).

Disease risk associated with the identified variants must be appropriately assessed especially that prophylactic mastectomy may also be considered for *ATM* and *CHEK2* mutations carriers depending on family history (Daly et al., 2017). Interestingly, in the current study we have identified a new homozygous likely pathogenic mutation in the *BLM* gene in a patient with multiple primary tumors, breast endometrial and colon cancers. Homozygous *BLM* mutations cause a rare autosomal recessive inherited disorder “Bloom Syndrome” that is characterized by chromosomal instability, immunodeficiency, and a predisposition to different types of malignancies, including breast and colon cancers (de Voer et al., 2015; Kluzniak et al., 2019). It was also shown that *BLM* may be a potential endometrial cancer predisposing gene which is consistent with our findings (Long et al., 2019). Other hereditary cancer syndromes predispose to cancer such as Lynch Syndrome. This latter is caused by pathogenic germline variants in Mismatch Repair Genes, *MLH1, MSH2, MSH6 or PMS2*, and predispose mainly to colorectal cancer and to other malignancies including endometrial, gastrointestinal, ovarian, pancreatic and skin cancers (Pande et al., 2012). In the current report, two variants of uncertain significance were identified in the *PMS2* gene, c.1004A>T and c.2186_2187del. This frameshift deletion is predicted to lead to the premature termination of the protein which in turn will alter the protein function. This same variant was previously reported in a homozygous state in a prostate cancer patient (Leongamornlert et al., 2014) and in a compound heterozygous state in cases with constitutional mismatch repair deficiency syndrome (CMMRD) (Bakry et al., 2014). In this CMMRD syndrome, homozygous carriers of *PMS2* gene mutation develop brain cancers in the first decade of life and 40% of patients may develop a second primary tumor (Ramchander et al., 2017). It was also previously described in a patient with colon cancer; yet, immunohistochemistry showed retained nuclear staining thus conflicting its pathogenicity (Haraldsdottir et al., 2017). In the current report, pathogenicity predictions for both *PMS2* variants were suggestive of a deleterious effect, however molecular dynamics simulation showed inconclusive results for c.1004A>T (N335I). Nevertheless, family history of both probands were consistent with lynch syndrome tumor spectrum which in turn may support the pathogenicity of the identified variants. Among the other MMR genes, we have identified a missense variant, c.728G>A in *MSH2* gene that seems to be likely pathogenic based on the findings of Kabbage et al. (2022). Indeed, this variant, which was described in a gastric cancer patient, appears to affect the MSH2-MLH1 complex as well as DNA-complex stability. Interestingly, both cases carrying this variant (current report & Kabbage et al., 2022) showed family history of cancer suggestive of lynch syndrome involving breast, gastric and ovarian malignancies which in turn may support the pathogenicity of the c.728G>A variant. In addition to MMR genes, we have identified a likely pathogenic splicing variant in *RAD50* gene that was predicted to lead to exon skipping (c.3036+5G>A). *RAD50* gene was associated with intermediate risk of developing breast cancer and updating the classification of this variant is important for better disease risk management (Damiola et al., 2014). Regarding *FANCC* and *FANCG* genes, identified variants were predicted to destabilize the protein structure and to modify its compactness. These alterations in protein structural integrity may have a significant effect on FANCC and FANCG activities. They could lead to loss of interactions among the Fanconi Anemia Core Complex alerting in consequence its important role in maintaining the genome activity. Mutations in the *FANCC* gene have been associated with an increased risk of developing breast cancer yet they seem to be rare compared to mutations in other DNA repair genes (Fang et al., 2020). Regarding *FANCG* gene, although it is usually included in the genetic diagnostic panels for hereditary breast cancer, its contribution to the genetic susceptibility of the disease is not well defined (Del Valle et al., 2020). Finally, considering *ERCC3* gene (c.2111C>T) *in silico* predictions revealed a potential deleterious effect yet stability and molecular dynamics simulation were not suggestive of a pathogenic effect. To better elucidate the functional impact of the studied variants *in vivo* or *in vitro* analysis, such as CRISPR/Cas9 genome editing tools, are required in order to validate the pathogenicity of these variants. This is crucial especially when variants are identified in unique families or when segregation analysis could not be performed. In the presence of strong evidence of pathogenicity, it is important to reclassify variants in order to avoid misinterpretation and to ensure appropriate patient care and adequate cancer risk assessment. Indeed, reviewing variant classification is of particular medical interest since carriers of pathogenic variants could undertake prophylactic surgery and may benefit from targeted therapy. Finally, and since variant reclassification has a significant impact on clinical management, physicians need to stay up to date with variant updates. Moreover, and as recommended by the National Comprehensive Cancer Network (Chiang et al., 2021), VUS carriers are invited to re-contact their genetics service providers after a few years asking for new updates regarding the pathogenicity of the identified variants.

## 5 Conclusion

In the current report, we have shown that variants with uncertain interpretations of pathogenicity may explain a part of the missing heritability of breast cancer in Tunisia. Our findings were supported by clinical and family history data along with segregation analysis, as well as *in silico* predictions and structural analysis results. This was particularly the case for *ATM* and *CHEK2* variants where we have found strong evidence arguing their pathogenicity. Therefore, for a better variant interpretation and classification, it is crucial to consider genomics data of African populations. It is also important to establish expert panel groups in understudied populations for variant curation and interpretation.

## Data Availability

The data presented in the study are deposited in the ClinVar repository, accession number SUB13085050.
