# Supplementary material for: Uncovering the clinical relevance of unclassified variants in DNA repair genes: a focus on BRCA negative Tunisian cancer families
Source: Front Genet. 2024 Jan 19;15:1327894. doi: 10.3389/fgene.2024.1327894 (PMC10834681; doi:10.3389/fgene.2024.1327894)
Supplement: Supplementary file 2 [file Image1.pdf]

Supplementary Material

1 Supplementary Figures

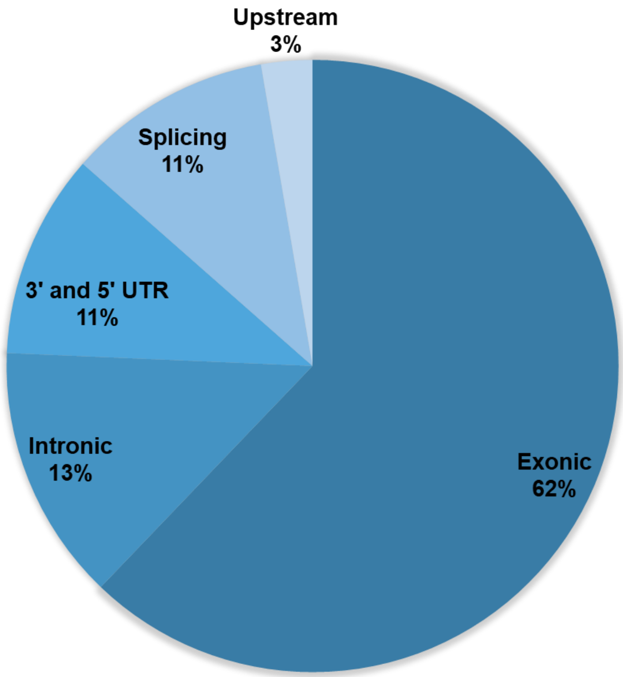

Supplementary Figure 1. *Distribution of unclassified DNA repair genes variants identified in this study*

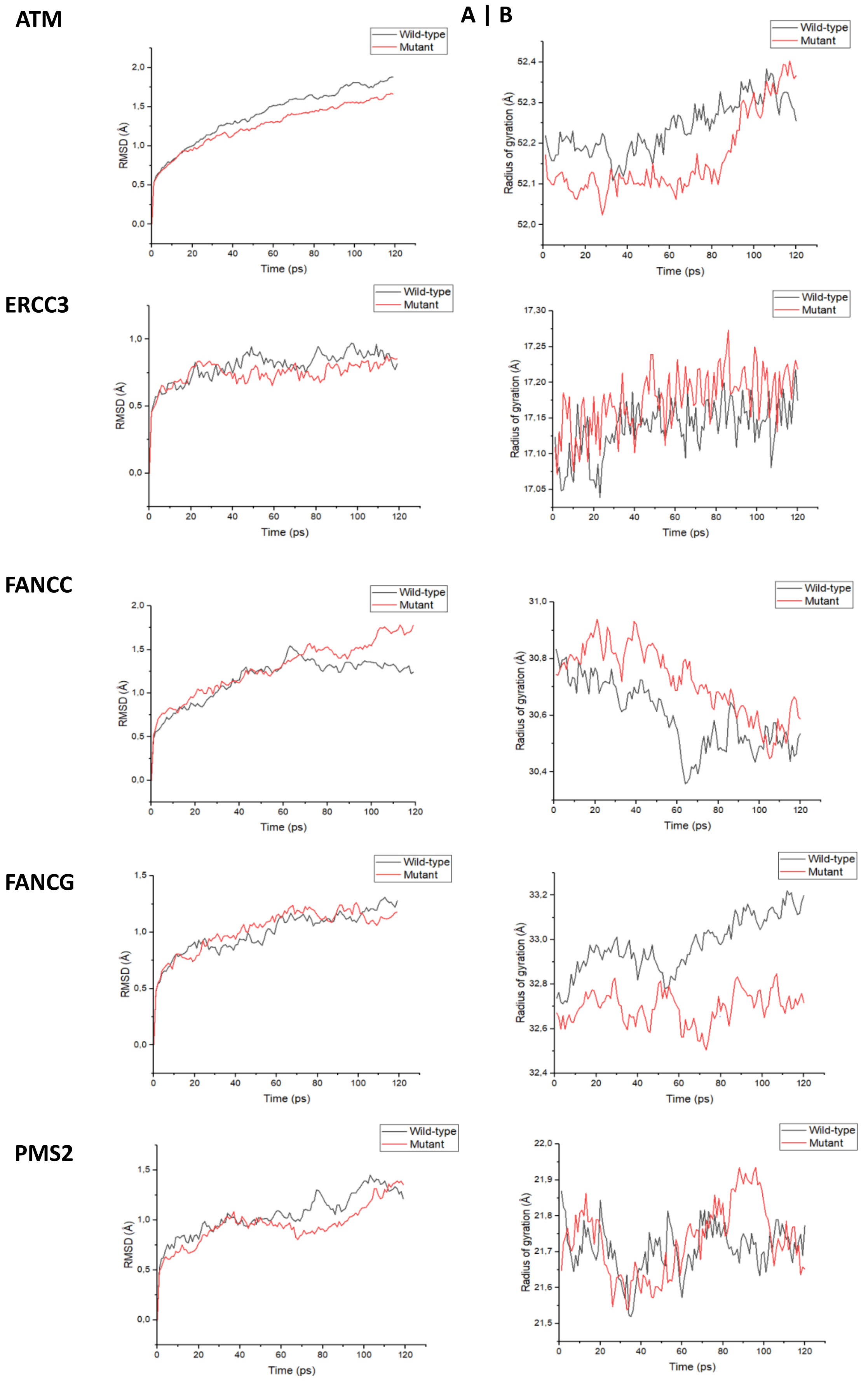

Supplementary Figure 2. *Molecular Dynamics simulations Analyses of ATM, FANCC, FANCG, ERCC3 and PMS2 proteins (A) Root mean square deviation (RMSD) and (B) Radius of gyration (RG) profiles of the native and mutant protein.*
